# Simple Techniques for Antegrade Guiding Catheter Engagement after Retrograde CTO Crossing

**DOI:** 10.1155/2020/7432831

**Published:** 2020-05-23

**Authors:** Gregor Leibundgut, Angelo Quagliana, Florian Riede, Heinz-Joachim Büttner

**Affiliations:** ^1^Department of Cardiology, Medizinische Universitätsklinik, Kantonsspital Baselland, Rheinstrasse 26, Liestal 4410, Switzerland; ^2^Cardiocentro Ticino, Lugano, Switzerland; ^3^Department of Health Promotion, Mother and Child Care, Internal Medicine and Medical Specialties, University of Palermo, Palermo, Italy; ^4^Klinik für Kardiologie und Angiologie II, Universitäts-Herzzentrum Freiburg-Bad Krozingen, Suedring 15, Bad Krozingen 79189, Germany

## Abstract

Interventional treatment of chronic total occlusions (CTOs) is nowadays counting on a wide span of procedural possibilities, and retrograde approaches are becoming more and more frequent as they warrant high success rates at the cost of a slightly higher incidence of donor vessel damage. Retrograde lesion crossing needs to be followed by procedural conversion to an antegrade approach to dilate and stent the lesion, and new techniques are being proposed to address this issue and achieve a safer recanalization of the vessel. In this context, we propose novel and simple techniques to antegrade guiding catheter engagement by the retrograde wire, enhancing the chances for procedural success.

## 1. Background

Chronic total occlusions (CTOs) account for almost 20% of all coronary lesions [1], and indications for CTO treatment have broadened over the last few years since their successful recanalization has been shown to improve angina, quality of life, and ventricular systolic function [2, 3]. The availability of more refined techniques and dedicated materials has increased procedural success up to 90% in experienced hands, according to the latest RCTs, at the cost of an acceptable risk of complications [4]. In this context, the possibility of CTO retrograde crossing through collateral circulations (CC) has represented a pivotal step forward in the achievement of such a high rate of successful procedures, since the distal end of the occlusion often offers a more funnel and softer entry point in comparison to the more blunted proximal one.

After the lesion has been retrogradely crossed by the use of support microcatheters and an extended armamentarium of dedicated coronary wires, further and specific technical skills are required to finalize the PCI. In fact, once the proximal true lumen of the occluded coronary artery has been reached retrogradely, whatever the technique used to cross the occlusion, a conversion to an antegrade procedure is usually recommended in order to reduce the risk of contralateral donor vessel dissection, spasm, or thrombus formation and finally allow stent deployment [5]. Procedural conversion implicates a preliminary engagement of the antegrade guiding catheter with the retrograde guidewire, which is usually attempted at the ostium of the occluded artery. Such attempts are not always successful, though, since their two tips need to be coaxial for the wire to enter the guide, while an eccentric exit point from the occlusion and heart cycle-related movements may interfere with their alignment. Ostial occlusions may even prevent successful guide placement at all.

In such cases, the snaring of a retrograde externalization wire from the antegrade guiding catheter has been considered the most suitable alternative by now. However, snaring is often time consuming, increases radiation exposure, and has an additional cost that can be avoided, making the whole procedure more cost-effective. The following paragraphs describe novel and simple techniques to engage the antegrade guiding catheter with the retrograde wire after unsuccessful ostial attempts, without the use of additional tools.

## 2. The Aortic Guide Engagement Technique (*Catch-It*)

**Figure 1:**
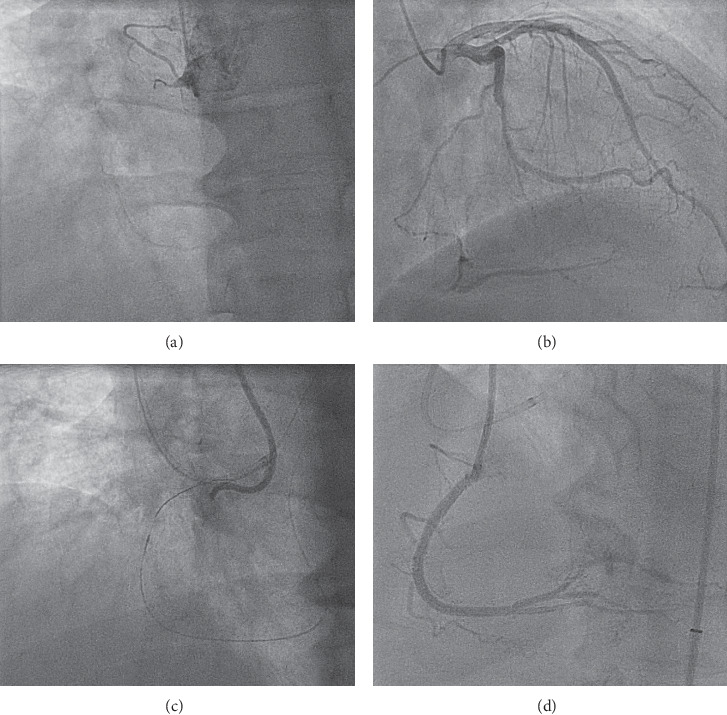
Case description. Key steps of the procedure: (a) RCA chronic total occlusion; (b) contralateral circulation from the donor vessel; (c) after successful CTO retrograde crossing, the retrograde wire cannot engage the antegrade guiding catheter for a lack of coaxiality; (d) final angiographic result.

The following case describes a new technique in the treatment of a CTO of the right coronary artery (RCA) by a retrograde approach with antegrade conversion (Figure 1). Double femoral access was established, with an AL1 guiding catheter for the ostial RCA and a 7F EBU 4.0 (Launcher, Medtronic) positioned at the ostium of the left main. After successful septal collateral crossing, a wire escalation strategy was chosen to cross the lesion. Finally, retrograde proximal cap puncture with a Gaia 3rd wire (Asahi Intecc) gave access to the true proximal lumen of the occluded RCA. Corsair (Asahi Intecc) microcatheter's advancement through the CTO was impossible, though. Several attempts to engage the antegrade AL 1 guiding catheter at the coronary ostium with the retrograde wire failed due to the lack of achievable coaxiality (Figure 1(c)). As a bailout solution, the retrograde wire was pushed out of the ostium of the RCA and advanced further up the ascending aorta, to attempt a novel *aortic guide engagement* maneuver we named “*Catch-it*”: after exiting the coronary ostium, the wire spontaneously follows the outer rim of the ascending aorta up to the arch. A 0.035″ wire can be inserted upside down inside the antegrade catheter while it is being retracted to the arch, in order to straighten its distal curve, improve its steerability (see Video 1 in Supplementary Material (available here)), and fix its position with the tip kept against the outer curve of the aortic arch. When the tips of the antegrade guiding catheter and the retrograde guidewire are aligned and minimally overlapped, a gentle turn on the guiding catheter can facilitate retrograde wire insertion into its distal orifice.

Once the engagement has been achieved, the retrograde wire can be advanced into the distal end of the guide for 3-4 centimeters, where it can be eventually trapped by the inflation of an antegrade balloon (previously described as *wire trapping* or *reverse anchoring balloon* [6, 7]). While keeping the balloon inflated, the AL 1 guide can be advanced towards the ostium of the RCA following the retrograde wire, which is simultaneously pulled contralaterally in order to prevent its looping in the ascending aorta (see Video 2 in Supplementary Material). When the antegrade guiding catheter has reached the ostial position, the retrograde microcatheter can be advanced across the CTO into the distal tip of the antegrade guide, supported by the trapped retrograde wire (see Video 3 in Supplementary Material).

**Figure 2:**
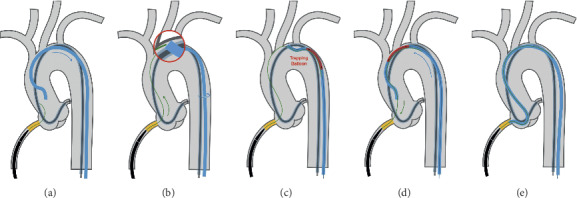
Aortic *catch-it* (*key-image should be largest*). Retrograde guidewire is unable to enter the antegrade guiding catheter. (a) Pullback the antegrade guide to the outer curve of the aortic arch (roof). (b) Advance the retrograde guidewire and position the tip close to the orifice of the retrograde guide in the aortic arch. Turn the antegrade guide to touch the retrograde wire. Move the guide back and forth to catch the wire (*catch-it*, red circle). (c) Trap the retrograde wire inside the antegrade guide with a 2.5/20 balloon at 12 atm. (d) Advance the antegrade guide while pulling the retrograde guidewire. (e) Place the antegrade guide at the RCA ostium.

**Figure 3:**
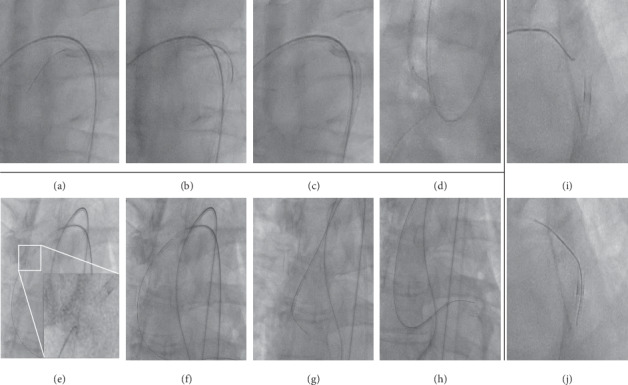
Aortic *catch-it* techniques: ascendens-arch-descendens. For general description of the method see the legend of Figure 1. *Catch-it* in the aortic arch: (a), (b), (c), and (d). *Catch-it* in the ascending aorta: (e), (f), (g), and (h). *Catch-it* in the descending aorta: (i) and (j).

The *catch-it* maneuver we described can be performed in the aortic arch, in the ascending aorta or in the descending aorta according to patient-specific anatomical conditions, in order to achieve the best position for the alignment of the antegrade catheter with the retrograde wire (Figures 2 and 3).

## 3. Antegrade Conversion

Contralateral exchange with the use of an externalization wire (>300 cm in length) is usually the technique of choice for the antegrade conversion of the procedure. The tip of such wires can be gently pushed inside the antegrade guide with the backup support of the retrograde microcatheter, or rather pulled backwards with an antegrade snare [6]. The high tension applied on the retrograde system to pull the retrograde wire out of the antegrade guiding catheter by a snare, as discussed previously, still carries some risk of donor vessel damage, though.

More recently, as an alternative to *externalization* [8], a so-called *antegrade microcatheter probing* has been proposed, by which the retrograde microcatheter, whose distal tip is positioned into the distal curve of the antegrade guiding catheter, is approached antegradely by a workhorse wire trying to enter its distal orifice [7]. This strategy allowed for the antegrade conversion in our case (see Video 4 in Supplementary Material), finally completed by the implantation of three drug-eluting stents with a good angiographic result of the RCA (Figure 1(d)) and without any procedural and postprocedural complications.

As far as antegrade conversion is concerned, several alternatives have been previously described in the literature and could provide helpful bailout solutions in case of failure of antegrade probing. A *rendezvous* could be attempted by advancing a second supporting microcatheter on the “probing” antegrade wire, supporting its insertion into the tip of the retrograde microcatheter [9]. The antegrade microcatheter should then be advanced on the wire, while the retrograde microcatheter is simultaneously retracted across the occlusion. Moreover, if heavily calcified ostial occlusions preclude retrograde microcatheter advancement across the proximal cap— notwithstanding the support of *wire trapping* techniques—a *tip-in* in the distal curve of the antegrade guide can help probing an antegrade microcatheter with the retrograde wire alone [6, 7, 10]. Alternatively, *reverse wire trapping* can be used to pull an antegrade snare through the occlusion and exchange it with an antegrade guidewire [11].

## 4. *Catch-It* in the Subclavian Artery: A Transradial Alternative

**Figure 4:**
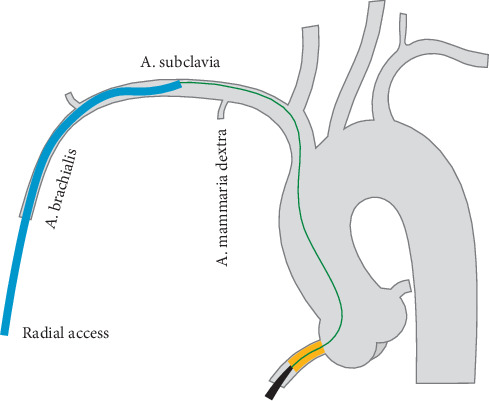
*Catch-it* in the subclavian artery. The antegrade guiding catheter's tip is kept against the roof of the subclavian artery. The 190 cm retrograde wire is gently advanced along the ascending aorta and the common trunk to reach the venue of the *catch*-it maneuver, where it can enter the distal tip of the antegrade guide. This step is followed by balloon trapping of the wire and by guide advancement to the RCA ostium, as described for the aortic *catch-it*.

Transradial approach to CTO treatment is becoming more frequent, driven by a significant reduction of bleeding complications and enhanced postprocedural patient comfort. In these cases, transfemoral tools and techniques need to be adapted to a different vascular anatomy. To perform an equivalent *catch-it* when the antegrade catheter has been advanced from the right radial access and the occlusion has been crossed retrogradely by a transfemoral approach, the right subclavian artery can provide optimal support for the antegrade catheter engagement by the retrograde wire. An antegrade guiding catheter enables its tip to be placed at the outer edge of the subclavian artery. A 190 cm long retrograde guidewire entering the common trunk and then the right subclavian artery can easily enter the antegrade guiding catheter, exploiting the backup support of the arterial roof (Figure 4). The engagement of the antegrade guide can be followed, as described above, by trapping of the retrograde wire (balloon [7] and snare [6]) and the subsequent advancement of the guide catheter through the ascending aorta towards the ostium of the occluded coronary artery. The procedure can then be converted to antegrade approach by *antegrade microcatheter probing* as in our procedure, with *rendezvous*, *tip-in*, *or reverse wire trapping* as helpful bailout alternatives [7, 9–11].

## 5. Externalisation through the Radial Sheath

**Figure 5:**
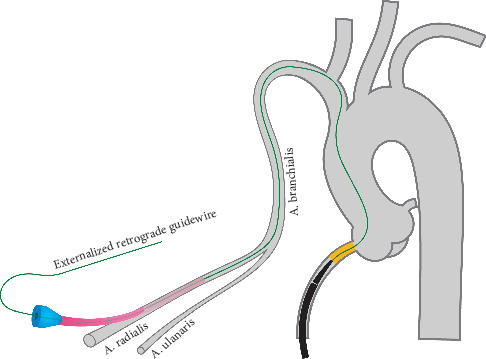
Externalisation through the radial sheath. In case of a subclavian *catch-it* failure, the antegrade guide can be retracted out of the radial sheath. A >300 cm retrograde wire is advanced up to the radial artery where it can easily enter the sheath, exploiting its flush apposition to the arterial wall. The tip of an antegrade catheter can facilitate wire externalization through the sheath valve without snaring it.

In case of failure of the *catch-it* maneuver in the subclavian artery, the 190 cm guidewire can be exchanged for a longer >300 cm externalization wire that can be advanced further until exiting the radial sheath. Since the sheath usually sits flush in the radial artery, the externalization wire naturally enters its distal orifice. To facilitate wire exit from the sheath, the tip of the antegrade guiding catheter can be inserted into its proximal valve (Figure 5).

## 6. Discussion

At the cost of a slightly higher risk of complications, such as periprocedural myocardial infarction, donor vessel injury, or perforation [12], retrograde approaches to CTOs are warranting an increasing rate of successful reperfusions in experienced hands, but evolving techniques require continuous operators' practice for skill maintenance and improvement. Beside procedural success, safety concerns have been addressed by the development of several techniques aiming at preserving the occluded and the contralateral vessel's integrity. A complete retrograde crossing of the lesion with the guidewire is not necessarily itself a prelude to vessel recanalization since retrograde wire advancement into the antegrade guiding catheter for procedural conversion is seldom unsuccessful, while a completely retrograde lesion dilatation and stenting through collateral circulation is nowadays considered harmful and accordingly discouraged.

We described simple alternatives to convert the procedure to a safer antegrade approach after successful retrograde lesion crossing, which may overcome the limits of the technique when the antegrade catheter is not engageable by the retrograde wire at the ostium of the occluded coronary artery. Such alternatives may be adapted both in case of transfemoral and transradial antegrade approaches, by changing the anatomical venue of the *catch-it* maneuver. The backup support of the aortic or the arterial roof is key to allow, in both cases, a perfect alignment of the retrograde guidewire and the antegrade guiding catheter and subsequently achieve wire insertion in its distal orifice. The following advancement maneuver of the antegrade guiding catheter to the coronary ostium is warranted by the support provided by balloon trapping of the retrograde guidewire [7], while simultaneously pulling back the retrograde wire contralaterally. The antegrade conversion of the procedure with the *catch-it* maneuver can avoid wire snaring in the aorta or brachiocephalic trunk and can be followed by a safe *antegrade microcatheter probing* [7], as in our case, alternatively replaced by a *rendezvous* [9] or a *tip-in* [10] attempt whenever unsuccessful, holding a good compromise between interventional success and patient's safety. As a bailout, retrograde externalization [8] through the radial sheath can still be performed by advancing a >300 cm dedicated wire, as we discussed above, and it requires no guide engagement maneuver (*catch-it* or snaring) at all.

## 7. Limitations and Advantages

Catheter manipulations inside the aortic root could be hindered by the presence of the retrograde guiding catheter and need to be performed with caution since an additional risk of vascular damage and debris cerebrovascular embolization cannot be eliminated. Similarly, extended manipulation with rather big snares may disrupt atherosclerotic lesion in the brachiocephalic trunk, significantly enhancing the risk of stroke.

The aortic *catch-it* maneuver allows to catch the retrograde wire with the antegrade guide without snaring and therefore without deforming its tip in the aortic lumen. In cases where the retrograde microcatheter is unable to cross the lesion, a *tip-in* of the retrograde guidewire into an antegrade microcatheter could be necessary and would be difficult with a deformed retrograde wire.

By contrast, a heavily calcified aortic arch, with large atherosclerotic lesions, may prevent the retrograde wire from entering the antegrade guiding catheter during an aortic *catch-it* attempt. Whenever the aortic engagement of the antegrade guide by the retrograde wire is hindered and proper alignment of the gear is difficult to achieve, switching to a subclavian approach for the engagement or to a wire externalization via the radial sheath may be an alternative option. Notably, some catheter shapes may be unsuitable for the aortic *catch-it*, but they still may work in a subclavian procedural setting. Definitions of all the techniques are given in Table 1.

## 8. Conclusions

Our proposals add to the wide span of dedicated techniques for CTO treatment, enhancing the chances of successful revascularisation while simplifying the procedure and reducing the risks of contralateral coronary artery damage.

## Figures and Tables

**Table 1:** Definition.

*Catch-it*	Antegrade guiding catheter engagement with the retrograde wire in a large vessel, such as the aorta or subclavian artery

*Tip-in* [10]	Retrograde wire insertion into the tip of an antegrade microcatheter, usually in the distal curve of the antegrade guiding catheter; usually when the retrograde microcatheter cannot pass the lesion and enter the antegrade guiding catheter

Rendezvous [9]	Antegrade guidewire, supported by an antegrade microcatheter, inserted into the retrograde microcatheter that has crossed the lesion. The two microcatheters meet in the guiding catheter

Antegrade microcatheter probing [7]	Intubation of the retrograde microcatheter with an antegrade workhorse wire in the distal curve of the antegrade guiding catheter

Wire trapping techniques [6, 7]	Trapping of the tip of the retrograde wire in the distal part of the antegrade guide by the inflation of an antegrade balloon (reverse anchoring balloon [7]) or antegrade snare [6]

Reverse wire trapping [11]	Antegrade microsnare grabbing the retrograde wire in the antegrade guiding catheter in order to pull the snare through the occlusion and exchange it with an antegrade guidewire

Externalisation [8]	Use of dedicated ≥300 cm externalization wires, entering the retrograde microcatheter and running through the occlusion to be pushed or pulled out contralaterally and allow the antegrade conversion of the procedure and entering an antegrade guide catheter

## Data Availability

Patient data, procedural data, and images are stored on the local hospital server and held available for 20 years.

## Supplementary Materials

Supplementary Materials
*Video 1*. Aortic *catch-it*: while the antegrade guide is kept against the aortic roof, the retrograde wire is advanced into its tip. *Video 2*. Wire trapping: the retrograde wire is trapped by a balloon inside the antegrade guide to provide support and advance the antegrade guide catheter down to the ostium of the RCA. *Video 3*. Microcatheter advancement into the antegrade guide: exploiting the support provided by the inflated trapping balloon, the retrograde microcatheter is advanced through the occlusion into the distal tip of the antegrade guide. *Video 4*. Antegrade microcatheter probing: advancement of an antegrade guidewire inside the retrograde microcatheter.
